# A Configurational Analysis of Small and Medium-Sized Enterprises’ Radical Innovations: The Perspective of Dynamic Capabilities

**DOI:** 10.3389/fpsyg.2021.784738

**Published:** 2022-01-18

**Authors:** Shuangshuang Tang, Shudi Liao, Lumeng Wang, Weijing Chen, Zhiwen Guo

**Affiliations:** ^1^School of Management, Huazhong University of Science and Technology, Wuhan, China; ^2^Business School, Hubei University, Wuhan, China; ^3^Hubei Center for Studies of Human Capital Development Strategy and Policy, Key Research Base of Humanities and Social Science of Hubei Province, Wuhan, China; ^4^College of Economics and Management, Huazhong Agricultural University, Wuhan, China

**Keywords:** environmental turbulence, absorptive capacity, SMEs, radical innovation, fsQCA

## Abstract

Adopting a configurational perspective, this study explored the pathways for small and medium-sized enterprises (SMEs) to achieve high levels of radical innovation. On the basis of dynamic capabilities theory, six causal conditions for radical innovation were identified at both external and internal levels—that is, environmental turbulence (i.e., technological and market turbulence) and absorptive capacity (i.e., knowledge base, explorative, transformative, and exploitative learning processes). The results of a fuzzy-set qualitative comparative analysis (fsQCA) of 82 Chinese SMEs identified four solutions for high radical innovation. The six causal conditions interacted interdependently and different combinations of these conditions were equally effective pathways for SMEs to achieve radical innovation. Hence, SMEs could generate radical innovation through flexibly allocating resources and capabilities based on the environmental circumstances. By using the fsQCA method, this study contributes to the related literature with an investigation of the complex causal relationship between environmental turbulence, absorptive capacity, and SMEs’ radical innovation. The results resolve some prior contradictory findings and provide new insights for future research. Other theoretical contributions, practical implications, and directions for future research are also discussed.

## Introduction

Small and medium-sized enterprises (SMEs) are important contributors to job creation and economic growth worldwide (Terziovski, 2010; Mamun et al., 2019). In the meantime, radical innovation, which refers to fundamentally new products or services that create discontinuities in technologies and/or the market (Chandy and Tellis, 1998; Garcia and Calantone, 2002; Alexander and van Knippenberg, 2014), is regarded as a critical source of competitive advantage and sustainable development (Christensen, 1997; Leifer et al., 2001). Although some scholars and practitioners view SMEs as the main drivers of radical innovation (Covin and Slevin, 1989; Hewitt-Dundas, 2006), some researchers find that SMEs tend to innovate less than large businesses (Schumpeter, 1942; Dewar and Dutton, 1986; Cáceres et al., 2011). Accordingly, various studies explore the different antecedents of SMEs’ radical innovation vs. large companies’ radical innovation (Vossen, 1998; Hewitt-Dundas, 2006; Prajogo and McDermott, 2014). However, instead of questioning whether different factors influence radical innovation by SMEs and large companies, we suggest that SMEs and large businesses follow different paths to achieve their radical innovation from a configurational perspective (Ragin and Fiss, 2008; Fiss, 2011).

Dynamic capabilities theory (Teece et al., 1997) states that winners in the rapidly changing business environment are firms that “can demonstrate timely responsiveness and rapid and flexible product innovation, coupled with the management capability to effectively coordinate and redeploy internal and external competences” (p. 33). In this vein, although SMEs are relatively less advantaged than large businesses in terms of access to material resources (e.g., economies of scale and technological, financial, and human resources) (Rothwell, 1985; Vossen, 1998; Arias-Aranda et al., 2001), they are more flexible and closer to the market and thus can respond faster to emerging technologies and customer needs (Laforet, 2013). Hence, SMEs have a unique strength in achieving radical innovation through flexibly reconfiguring their limited resources depending on the requirements of the business environment (Rothwell, 1985; Hewitt-Dundas, 2006; Prajogo and McDermott, 2014). Therefore, we are interested in exploring how SMEs achieve high radical innovation when they experience resource constraints.

According to dynamic capabilities theory, absorptive capacity, defined as firms’ ability to “identify, assimilate, and exploit knowledge from the environment” (Cohen and Levinthal, 1989, p. 569), is essential for firms to generate radical innovation in the turbulent business environment (Van den Bosch et al., 1999; Lichtenthaler and Lichtenthaler, 2009). Absorptive capacity is operationalized as a knowledge base [e.g., research and development (R&D) intensity or patents] and then reified into three learning processes (i.e., exploratory, transformative, and exploitative learning) by which firms utilize external knowledge to create new knowledge (Cohen and Levinthal, 1989; Lane et al., 2006). A firm’s knowledge base and these three learning processes mutually complement and reinforce each other, and they constitute the firm’s absorptive capacity (Zahra and George, 2002; Roberts et al., 2011; Carlo et al., 2012). However, it is difficult for SMEs to simultaneously invest in R&D and all three learning processes because of their resource constraints (Gupta et al., 2006). Instead, in order to achieve radical innovation, SMEs should capitalize on their organizational flexibility and adjust their innovative strategies to capture the technological and market turbulence (Covin and Slevin, 1989; Hewitt-Dundas, 2006).

In addition, SMEs’ resource constraints increase their vulnerability to external changes (Covin and Slevin, 1989; Bodlaj and Cater, 2019). Thus, the business environment plays a particularly important role in SMEs’ innovative processes (Prajogo and McDermott, 2014). Many studies use a contingency perspective to examine the moderating effects of environmental turbulence in the relationship between absorptive capacity and radical innovation (Jansen et al., 2006; Teece, 2007; Lichtenthaler, 2009). However, as indicated by Lane et al. (2006), environmental turbulence determines “the incentives for investing in absorptive capacity” (p. 857) and can also be an antecedent of absorptive capacity and radical innovation from a process perspective (Cohen and Levinthal, 1989; Van den Bosch et al., 1999; Bodlaj and Cater, 2019). Therefore, the causal relationship between environmental turbulence, absorptive capacity, and SMEs’ radical innovation is complex. Partially because of the limitations of symmetric methods (Douglas et al., 2020), few empirical research studies capture this causal complexity or the multidimensional nature of absorptive capacity (Todorova and Durisin, 2007; Lichtenthaler, 2009; Ferreras-Méndez et al., 2016).

The fuzzy-set qualitative comparative analysis (fsQCA) method (Ragin, 1987, 2000) is used in this study because fsQCA assumes that many causal conditions (i.e., independent variables) affect an outcome interdependently and that different configurations (i.e., combinations of causal conditions) can equivalently lead to the same outcome (Ragin and Fiss, 2008; Rihoux and Ragin, 2009; Fiss, 2011; Pappas and Woodside, 2021). Therefore, by using fsQCA, we aim to make the following contributions. First, from a configurational perspective, we can explore different possible solutions that explain the relationship between environmental turbulence, absorptive capacity, and SMEs’ radical innovation and thus provide some fresh directions for further research into SMEs’ radical innovation. Second, fsQCA identifies causal asymmetries—that is, conditions can be related, unrelated, or even inversely related to the outcome in different configurations (Meyer et al., 1993; Woodside, 2013). So the results of this study can help resolve previously contradictory findings in the relevant research literature. Third, we extend the absorptive capacity research literature by simultaneously examining the effects of the knowledge base, explorative, transformative, and exploitative learning processes in one theoretical model and their complementarity.

## Theoretical Background

### Small and Medium-Sized Enterprises’ Radical Innovation

Innovation is “the intentional introduction and application within a role, group or organization of ideas, processes, products or procedures, new to the relevant unit of adoption, designed to significantly benefit the individual, the group, organization or wider society” (West and Farr, 1990, p. 9). The degree of newness distinguishes radical innovation from incremental innovation (Dewar and Dutton, 1986). Incremental innovation refers to simple improvements or minor extensions to current products or processes (Dewar and Dutton, 1986; McDermott and O’Connor, 2002), and radical innovation represents fundamental changes in technology and clear departures from existing products, processes, or services (Dewar and Dutton, 1986; Chandy and Tellis, 1998). Radical innovation cannot only better satisfy customers’ needs but also create substantially new benefits for customers (Chandy and Tellis, 1998; Atuahene-Gima, 2005). In addition, radical innovation can offer significant improvements (e.g., ≥ 5-fold) in organizational performance or significant reductions (≥30%) in cost (Leifer et al., 2001). Thus, for opportunity-focused SMEs, the generation of radical innovation is an important way to break the *status quo*, obtain a competitive advantage, and guarantee growth (Christensen, 1997; Bodlaj and Cater, 2019).

Scholars have paid close attention to the determinants of radical innovation, among which firm size has drawn strong interest; however, the results are controversial (Ettlie et al., 1984; Dewar and Dutton, 1986; Covin and Slevin, 1989; Hewitt-Dundas, 2006). One reason may be that these researchers examine the determinants of SMEs’ radical innovation vs. large firms’ radical innovation from different perspectives (Prajogo and McDermott, 2014). According to the resource-based view of the firm and Schumpeter’s classic arguments on creative accumulation, some researchers suggest that large firms possess more financial and technological resources, enjoy economies of scale and scope, and thus have a greater advantage over SMEs in adopting radical innovation (Schumpeter, 1942; Grant, 1991; Arias-Aranda et al., 2001). However, other researchers demonstrate from a behavioral perspective that large firms are more bureaucratic, tend to get trapped in their core competences, and react slowly to technological changes or changing customer needs (Levinthal and March, 1993; Mitchell and Singh, 1993; Damanpour and Wischnevsky, 2006). These behavioral constraints make large firms less innovative than SMEs in dynamic environments (Hewitt-Dundas, 2006). In contrast, SMEs are comparatively disadvantaged in terms of resources, but they are superior in their behavioral aspects—that is, they are more flexible, efficient, and motivated (Rothwell, 1985; Vossen, 1998; Prajogo and McDermott, 2014).

Adopting a configurational perspective, we argue that what matters here are not only the different determinants of radical innovation between SMEs and large businesses but also the different pathways between them to achieve high radical innovation (Slater et al., 2014; Douglas et al., 2020). Radical innovation is a complex business phenomenon characterized by high risks and uncertainties, and the innovative process is full of unpredictable challenges (Alexander and van Knippenberg, 2014; Colombo et al., 2017). Thus, resources and capabilities are essential for both SMEs’ and large businesses’ radical innovation (Chang et al., 2012; Zhou and Li, 2012; Tiberius et al., 2021). Firms have their own pathways to achieve radical innovation through different configurations of environmental factors and internal and external resources and capabilities (Poorkavoos et al., 2016). The strength of organizational flexibility allows SMEs to overcome the constraints of material resources by adapting their limited resources and capabilities to the changing demands; thus, SMEs are expected to be better positioned to generate radical innovation in the turbulent business environment (Covin and Slevin, 1989; Hewitt-Dundas, 2006; Laforet, 2013).

### Absorptive Capacity and Radical Innovation

Teece et al. (1997) defines dynamic capabilities as “the firm’s ability to integrate, build, and reconfigure internal and external competences to address rapidly changing environments” (p. 34) and proposes that firms should continually renew their competences to achieve and maintain new forms of competitive advantages (Leonard-Barton, 1992; Teece et al., 1997). Similarly, given the growing complexity and uncertainty in the business environment, Chesbrough’s open innovation model suggests that to innovate successfully, firms should shift their focus from spending on internal R&D to searching for and acquiring external knowledge and expertise outside the organization’s boundaries (Chesbrough, 2003, 2006; Laursen and Salter, 2006). Accordingly, as an essential component of its dynamic capabilities, a firm’s absorptive capacity—the ability to recognize the potentially valuable external knowledge, assimilate it, and apply the assimilated knowledge to commercial ends—is critical for the firm to take advantage of externally held knowledge to generate radical innovation (Cohen and Levinthal, 1989; Eisenhardt and Martin, 2000; Lane et al., 2006; Lichtenthaler and Lichtenthaler, 2009). The research literature shows that absorptive capacity can facilitate firms’ radical innovation (Van den Bosch et al., 1999; Ritala and Hurmelinna-Laukkanen, 2013; Flor et al., 2018).

Absorptive capacity was initially put forward by Cohen and Levinthal (1989), who use the term to describe a firm’s ability to create new knowledge by identifying, assimilating, and exploiting knowledge from the external environment. Absorptive capacity has since become one of the most important constructs in the organizational and management research literature (Lane et al., 2006). Although originally conceptualized as a firm’s ability, absorptive capacity is considered to be a firm’s current knowledge base and is empirically equated with the firm’s R&D spending or patents (Cohen and Levinthal, 1989; Mowery et al., 1996; Ahuja and Katila, 2001). Later, some studies redefine absorptive capacity from the perspective of the firm’s dynamic capabilities (e.g., Dyer and Singh, 1998; Lane and Lubatkin, 1998; Van den Bosch et al., 1999). Among these, Zahra and George’s (2002) reconceptualization is widely used. They put emphasis on “a set of organizational routines and processes by which firms acquire, assimilate, transform, and exploit knowledge (p. 186)” and distinguish between potential (knowledge acquisition and assimilation) and realized (knowledge transformation and exploitation) capacity (Zahra and George, 2002).

Lane et al. (2006) further integrate the insights from previous studies and extend the concept from a more process-oriented perspective. They argue that the benefits of absorptive capacity depend on the underlying exploratory, transformative, and exploitative learning processes that allow the firm to consciously create, expand, or modify its knowledge base (Eisenhardt and Martin, 2000; Ferreras-Méndez et al., 2016; Forés and Camisón, 2016). Exploratory learning refers to the process of recognizing and acquiring valuable new knowledge from the external environment, and exploitative learning involves transforming and applying the acquired external knowledge into commercial outputs (Levinthal and March, 1993; Lane et al., 2006; Ferreras-Méndez et al., 2016). These two learning processes also correspond to potential and realized absorptive capacity (Zahra and George, 2002; Lichtenthaler, 2009). Transformative learning links exploratory and exploitative learning is—that is, the firm maintains valuable knowledge and reactivates related knowledge when needed (Garud and Nayyar, 1994; Lane et al., 2006; Ferreras-Méndez et al., 2016). Thus, a firm’s absorptive capacity consists of (1) its knowledge base and (2) the three learning processes through which the firm utilizes external knowledge (Cohen and Levinthal, 1989, 1990; Lane et al., 2006).

The knowledge base represents a firm’s most pivotal and unique resource for radical innovation (Zhou and Li, 2012). The knowledge base determines whether a firm can accurately predict technological trends and react to the emerging opportunities in time (Cohen and Levinthal, 1994; Teece, 2007). It influences not only the breadth of external knowledge searching and recognizing but also the depth of knowledge that a firm can understand (Mowery et al., 1996; Ahuja and Katila, 2001; Lane et al., 2006). Acquired and assimilated knowledge from external sources through the exploratory, transformative, and exploitative learning processes in turn eases the scarcity of internal knowledge resources and enriches the firm’s knowledge base (Cohen and Levinthal, 1990; Van den Bosch et al., 1999). Thus, a firm’s knowledge base and the three learning processes interact in a complex way and interdependently affect the creation of radical innovation (Todorova and Durisin, 2007; Carlo et al., 2012; Ritala and Hurmelinna-Laukkanen, 2013). However, because of firms’ internal resource constraints, high levels of R&D spending and the three learning processes may not coexist in most firms, especially in SMEs (Gupta et al., 2006). Instead, firms should constantly balance their investments in R&D and the learning processes to address the dynamically changing environment and achieve radical innovation (Teece et al., 1997; Tang et al., 2020).

### Environmental Turbulence and Radical Innovation

In proposing the absorptive capacity construct, Cohen and Levinthal (1989, 1990) highlight the role of the environmental context in determining firms’ investment in their absorptive capacity (Van den Bosch et al., 1999; Lane et al., 2006). The innovation research literature also considers the external environment as a primary stimulus for firms to generate radical innovation (Damanpour and Wischnevsky, 2006; Prajogo and McDermott, 2014). Based on dynamic capabilities theory, the fast-moving business environment exposes firms’ current products or services to the risk of being made obsolete at any time (Teece, 2007; Lichtenthaler, 2009). Therefore, in general, with increasing dynamism and hostility in the environment, firms’ emphasis will shift from incremental innovation to radical innovation that deviate from existing technologies and/or markets (Dewar and Dutton, 1986; Jansen et al., 2006; Droge et al., 2008). While resource scarcity makes SMEs more vulnerable to environmental turbulence, it also forces SMEs to become more external-oriented and more sensitive to environmental changes (Bodlaj and Cater, 2019). As a result, SMEs seek to improve their innovativeness under the prevailing conditions to stay competitive in the turbulent business environment (Covin and Slevin, 1989; Uzkurt et al., 2012).

Environmental turbulence includes both technological and market turbulence (Jaworski and Kohli, 1993; Lichtenthaler, 2009). Technological turbulence refers to “the rate of technological change” (Jaworski and Kohli, 1993, p. 57). Firms operating in a highly technologically turbulent environment must continually explore new knowledge and technologies to increase their opportunities to generate radical innovation, which can help them obtain first-mover advantages and sustain their growth (Lichtenthaler, 2009; Bodlaj and Cater, 2019). In their meta-analysis, Huang and Tsai (2014) identify a positive relationship between technological turbulence and product innovativeness. Market turbulence refers to “the rate of change in the composition of customers and their preferences” (Jaworski and Kohli, 1993, p. 57). In turbulent markets, firms’ products and services must be constantly modified, updated, or even replaced to better meet their customers’ changing needs (Jaworski and Kohli, 1993; Chandy and Tellis, 1998). Compared with larger firms, SMEs interact more closely with their customers and can understand and respond more quickly to their customers’ inquiries (Covin and Slevin, 1989; Salavou et al., 2004). Using data from SMEs in Turkey, Uzkurt et al. (2012) find positive effects for both technological and market turbulence on the SMEs’ innovativeness. Similarly, Bodlaj and Cater (2019) demonstrate a direct positive impact of market turbulence on SMEs’ innovativeness.

Considering the relationship between environmental turbulence, absorptive capacity, and SMEs’ radical innovation, some scholars view environmental turbulence as a moderating factor (e.g., Lichtenthaler, 2009), whereas others regard it as an antecedent and examine the direct and indirect effects of technological and market turbulence on SMEs’ radical innovation (e.g., Uzkurt et al., 2012; Bodlaj and Cater, 2019). However, as Slater et al. (2014) suggest, these direct effects models provide linear additive explanations but underestimate the interdependence between these causal conditions when influencing radical innovation. Likewise, Douglas et al. (2020) show that the traditional quantitative methods that dominate the literature do not sufficiently deal with the heterogeneity of complex business phenomena. Thus, by answering calls to use the fsQCA method to reveal a finer-grained understanding of the complexity of radical innovation (Fiss, 2011; Ganter and Hecker, 2014; Douglas et al., 2020), we use fsQCA to explore the various pathways by which SMEs can achieve high radical innovation. From a dynamic capabilities perspective, we identify six causal conditions at both external and internal levels: technological turbulence, market turbulence, firms’ knowledge base, explorative learning, transformative learning, and exploitative learning.

## Materials and Methods

### Data and Sample

The data were collected from 82 SMEs located in Wuhan, the largest city in central China. By 2020, more than 6,000 high-tech enterprises have been operating in Wuhan. We asked some top-level managers of SMEs who participated in our MBA program to recommend other top-level managers of SMEs to participate in this research. Like most fsQCA studies in management (e.g., Fiss, 2011; Poorkavoos et al., 2016), we designed a cross-sectional questionnaire to obtain the data for the conditions that we explored in this study and the background information of the SMEs and the managers. A total of 209 questionnaires were sent to top-level managers of SMEs through an online survey, and 82 were returned (39.2% response rate). The sizes of the sampled SMEs ranged from 9 to 500 employees, with a median of 80 employees. The ages of the sampled SMEs ranged from 1 to 42 years, with a median of 14 years. The SMEs operated in various industries, such as machinery and equipment manufacturing, information technology, and construction (see Table 1 for a detailed industry distribution).

**TABLE 1:** Industry distribution of the sampled SMEs.

Industry	n
Machinery and equipment manufacturing	17
Information technology	10
Services	8
Construction industry	7
Electric engineering	6
Trade industry	6
Medicine and health	6
Consumer products	4
Finance	4
Real estate industry	3
Education	3
Environmental protection	3
Chemical products	2
Logistics and supply chain	2
Agricultural industry	1
Total	82

### Measurement

We used the 9-item scale developed by Poorkavoos et al. (2016) to measure SMEs’ *radical innovation*. The respondents were asked to rate their performance compared with their competitors operating in the same industry sector to compare the data at the cross-industry level (Oke, 2007; Poorkavoos et al., 2016). Following Bodlaj and Cater (2019), *technological turbulence* and *market turbulence* were measured by three items each from the widely used scales developed by Jaworski and Kohli (1993). *Knowledge base* is usually considered as R&D intensity, i.e., the ratio of firms’ annual R&D expenditure to their sales (Cohen and Levinthal, 1989; Laursen and Salter, 2006). Because of difficulties in collecting objective data for R&D expenditure and sales, we used the proportion of firms’ R&D employees to their total number of employees as a proxy of the firms’ R&D intensity. The proportion of firms’ R&D employees is also one of the important indicators of firms’ R&D capabilities (Visalakshi and Sandhya, 1997). Finally, the *exploratory*, *transformative*, and *exploitative learning processes* were measured using the scale developed by Ferreras-Méndez et al. (2016). Exploratory learning captures firms’ activities in recognizing and assimilating valuable external knowledge, transformative learning comprises the activities of maintaining and reactivating the firms’ relative knowledge, and exploitative learning refers to the activities of transmuting and applying new and existing knowledge into commercial products (Garud and Nayyar, 1994; Jansen et al., 2005; Ferreras-Méndez et al., 2016). The scale consists of 18 items, and each learning process was assessed using 6 items. The complete measurement scales used in this study are presented in Appendix. All items were translated to Chinese using a back-translation procedure (Brislin, 1986) and were measured using a 7-point Likert-type scale (where 1 = “strongly disagree” and 7 = “strongly agree”). Table 2 shows the reliability test of the measurement.

**TABLE 2:** Reliability test of the measurement.

Condition	Factor loadings*	Composite reliability	Cronbach’s alpha
Radical innovation (9 items)	0.580 ∼ 0.809	0.926	0.908
Technological turbulence (3 items)	0.883 ∼ 0.946	0.939	0.902
Market turbulence (3 items)	0.788 ∼ 0.929	0.913	0.856
Exploratory learning (6 items)	0.781 ∼ 0.893	0.926	0.903
Transformative learning (6 items)	0.680 ∼ 0.812	0.897	0.859
Exploitative learning (6 items)	0.695 ∼ 0.873	0.927	0.903

*All the factor loadings were significant at p < 0.05.

### Analytical Technique

**FIGURE 1:**
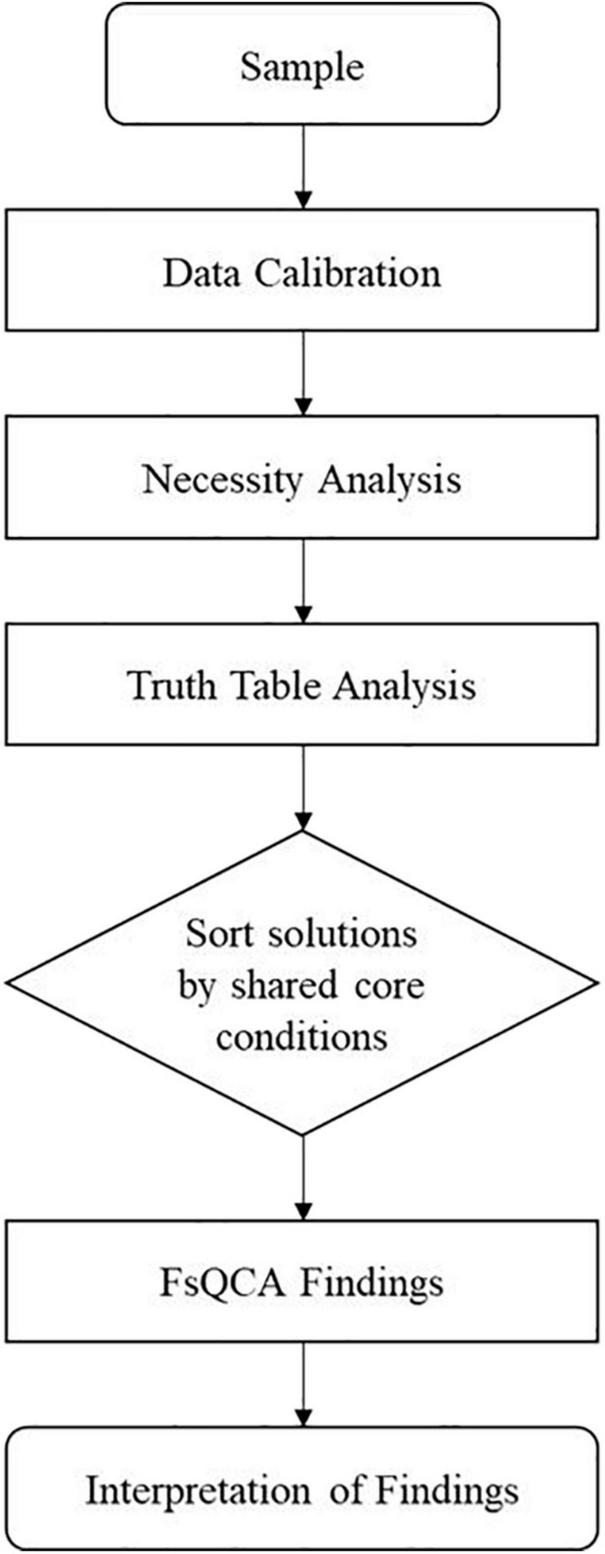
The flowchart of the fsQCA method.

The data were analyzed using fsQCA 3.0 software. Unlike variance-based methods, fsQCA is grounded in set theory and analyzes data at the case level (Ragin, 2006; Ragin and Fiss, 2008; Pappas and Woodside, 2021). Each causal and outcome condition is regarded as a fuzzy set, and all of the collected data should be transformed into fuzzy sets through a calibration process (Schneider and Wagemann, 2012). After calibration, all of the scores of the conditions range from 0 to 1, with 0 representing full non-membership, 0.5 representing the crossover point, and 1 representing full membership (Ragin, 2000, 2008). By computing each case’s degree of membership in the causal and outcome condition sets, fsQCA can deal with cases of different sample sizes and data of different types. Therefore, this analytical technique goes beyond qualitative and quantitative strategies (Ragin, 1987; Rihoux and Ragin, 2009; Pappas and Woodside, 2021). A flowchart of the fsQCA method utilized in this study is presented in Figure 1 and each step will be explained in details in the following part.

## Results

### Calibration

We used a direct method for calibration and chose the upper quartile, median, and lower quartile values commonly used as the thresholds for the three points of membership to calibrate the SMEs’ radical innovation, the technological and market turbulence, and the three learning processes (Ortiz de Guinea and Raymond, 2020; Pappas and Woodside, 2021). To calibrate SMEs’ R&D intensity, we set 20, 10, and 0% as the thresholds because one of the criteria for a firm to be certificated as a high-tech enterprise in China is that the proportion of firms’ R&D employees should not be lower than 10%. Following Fiss (2011), a constant of 0.001 was added to all scores below 1 after calibration to avoid values of 0.5, which cannot be analyzed by the fsQCA 3.0 software (Ragin, 2008; Wagemann et al., 2016). The descriptive statistics and the calibration thresholds for the outcome and the causal conditions are shown in Table 3.

**TABLE 3:** Descriptive statistics and calibration thresholds.

Condition	Mean	SD	Fuzzy-set calibration		
			Full membership	Crossover	Full non-membership
Radical innovation	4.41	1.03	5.00	4.28	3.78
Technological turbulence	4.81	1.39	6.00	5.00	4.00
Market turbulence	4.39	1.34	5.25	4.67	3.67
R&D intensity	0.15	0.15	0.20	0.10	0.00
Exploratory learning	5.35	0.94	6.00	5.50	4.83
Transformative learning	5.60	0.70	6.00	5.50	5.04
Exploitative learning	5.22	0.90	6.00	5.25	4.83

### Necessity Analysis

To reveal the complex causal relationships between causal conditions and an outcome of interest, fsQCA views each case as a configuration of causal conditions and indicates the necessary and sufficient conditions/configurations of the outcome through a comparative analysis of cases (Ragin, 2000; Rihoux and Ragin, 2009). The necessity analysis should be conducted before the sufficiency analysis to detect the necessary condition(s) in advance (Schneider and Wagemann, 2012). A condition is considered as a necessary condition for the outcome when the outcome set is a subset of the condition set—that is, the outcome cannot be present without the presence of the condition (Caramani, 2008). Table 4 presents the results of necessity analysis. The consistency score indicates the proportion of cases whose membership in the condition set is greater than their membership in the outcome set (Rihoux and Ragin, 2009). When it is above 0.9, the condition can be identified as a necessary condition (Ragin, 2008; Schneider, 2018). Therefore, in this study, no single condition was a necessary condition for the SMEs to achieve high radical innovation.

**TABLE 4:** Necessity analysis.

Causal condition	High radical innovation	
	Consistency	Coverage
Technological turbulence	0.588	0.592
Market turbulence	0.571	0.573
R&D intensity	0.705	0.620
Exploratory learning	0.685	0.672
Transformative learning	0.699	0.622
Exploitative learning	0.683	0.708

### Sufficiency Analysis

Sufficiency analysis is conducted through the generation of a truth table with the presence of SMEs’ radical innovation (i.e., high levels of radical innovation) as the outcome. The truth table contains 2^k^ rows, where k equals the number of causal conditions and each row represents a possible configuration of the causal conditions to the presence of the outcome (Ragin, 2008). We identified six causal conditions in this study; therefore, the truth table included 64 rows with 40 observed configurations and 24 logical remainders, which are the logically possible configurations without empirical instances (Ragin, 2008). The truth table is then sorted by frequency and consistency (Ragin, 2008). Frequency refers to the number of cases reflected in each configuration. Given the sample size of this study, we set the frequency threshold at 2 (Fiss, 2011). Consistency in the sufficiency analysis refers to the extent to which the configuration constitutes the subset of the outcome set, i.e., the extent to which the configuration is a sufficient configuration for the outcome (Ragin, 2006; Rihoux and Ragin, 2009). As recommended by Ragin (2008), the consistency threshold was set at 0.75. The proportional reduction in inconsistency (PRI) consistency is also considered in fuzzy sets analysis, and the threshold was set at 0.70 (Greckhamer et al., 2018).

The truth table analysis makes counterfactual reasoning about logical remainders and provides three types of solutions: complex, parsimonious, and intermediate solutions (Ragin, 2000, 2008). Intermediate solutions and parsimonious solutions are recommended for interpreting the results (Fiss, 2011). Table 5 shows the results of the truth table analysis for the presence of SMEs’ radical innovation. Using the notation from Ragin and Fiss (2008), the black and crossed-out circles represent the presence and absence of a condition, respectively. The blank space indicates that whether the condition is present or absent is indifferent to the outcome. Conditions appearing in both intermediate and parsimonious solutions are called “core conditions” and are marked with a large circle, whereas the conditions appearing only in intermediate solutions are called “peripheral conditions” and are marked with a small circle (Ragin and Fiss, 2008). The “coreness” represents “the strength of the evidence relative to the outcome” (Fiss, 2011, p. 403). The solutions were sorted by their shared core conditions (Fiss, 2011). Coverage refers to the extent to which the configuration is the only solution leading to the outcome and thus reflects the importance of the configuration (Ragin, 2006).

**TABLE 5:** Configurations for SMEs’ high radical innovation.

Configuration	Solution			
	1	2	3a	3b
*Environmental turbulence*				
Technological turbulence	•	•	⨂	⨂
Market turbulence		🌑	⊗	⊗
*Absorptive capacity*				
R&D intensity	•	🌑		•
Exploratory learning		•	•	⨂
Transformative learning	•	🌑	•	⨂
Exploitative learning	•		🌑	🌑
Consistency	0.754	0.786	0.871	0.927
Raw coverage	0.343	0.270	0.165	0.103
Unique coverage	0.100	0.049	0.092	0.050
Overall solution consistency	0.806			
Overall solution coverage	0.540			

*The black circles* (🌑) *represents the presence of a condition, the crossed-out circle* (⊗) *means the absence of a condition, and the blank space indicates a “don’t care” situation. Large and small circles represent core conditions and peripheral conditions, respectively.*

This study identified four pathways that were sufficient for SMEs to achieve high radical innovation. The overall consistency was 0.806 and the overall coverage was 0.540. Solution 1 includes the presence of technological turbulence, R&D intensity, transformative learning, and exploitative learning as a configuration for high radical innovation. The outcome would not be affected whether market turbulence and explorative learning were present or absent. This solution highlights the importance of developing and maintaining the firm’s own core competence. As mentioned, the firm’s knowledge base is its most unique resource for standing out from others and obtaining competitive advantages (Zhou and Li, 2012). Although dynamic capabilities theory suggests that explorative learning is more important in turbulent business environments (Jansen et al., 2006; Teece, 2007), explorative learning requires the input of time and financial resources and firms’ networking and managerial capabilities (Cohen and Levinthal, 1990; Oerlemans et al., 2013). For SMEs whose resources and capabilities are naturally constrained, Solution 1 suggests that SMEs should prioritize the allocation of their limited resources to R&D, transformative learning, and exploitative learning.

Solution 2 indicates the configuration of the presence of technological and market turbulence, R&D intensity, exploratory learning, and transformative learning, with market turbulence, R&D intensity, and transformative learning as the core conditions. In this solution, exploitative learning is indifferent, which shows that when both technology and customer needs change rapidly, internal exploitation is less valued. Instead, the need for exploratory learning increases, which echoes previous research into dynamic capabilities (Jansen et al., 2006; Teece, 2007). In addition, solution 2 emphasizes the roles that R&D intensity and transformative learning play in a turbulent environment. On the one hand, it becomes harder for firms to recognize and acquire potentially valuable knowledge under highly uncertain conditions, which requires SMEs to flexibly cope with external changes by maintaining and expanding a large knowledge base (Taylor and Greve, 2006; Teece, 2007). On the other hand, external knowledge acquisition might be insufficient under such circumstances, which poses challenges for transformative learning (Marsh and Stock, 2006). Transformative learning is especially significant in dynamic environments because it takes time—sometimes years—for customers to accept new technology and products. Thus, the assimilated external knowledge may need to be maintained for a long time until before it can be applied to commercial outputs for radical innovation (March, 1991).

Solutions 3a and 3b indicate two pathways to high radical innovation under stable environments, where technological and market turbulence are both absent. These pathways share the core conditions of the absence of market turbulence and the presence of exploitative learning. These two solutions show that in a relatively stable environment, SMEs can capitalize on exploitative learning to achieve radical innovation (Gupta et al., 2006). Exploitative learning is the process of applying the knowledge to match the markets (Lenox and King, 2004; Smith et al., 2005). SMEs are closer to their customers; therefore, they could perform better at understanding and fulfilling their customers’ needs (Salavou et al., 2004). Solutions 3a and 3b also demonstrate that high levels of exploratory and transformative learning processes and a high level of R&D intensity can be substitutes for each other. This result verifies that radical innovation can emerge from a knowledge base either developed by the firm itself or drawn entirely from external sources (Hill and Rothaermel, 2003; Zhou and Li, 2012). The raw coverage scores for solutions 3a and 3b were smaller than those for solutions 1 and 2, which shows that SMEs will be more motivated to introduce radical innovation in a turbulent environment.

We also conducted a sufficiency analysis for the absence of radical innovation (i.e., low to medium levels of radical innovation). The frequency and PRI consistency thresholds were still set at 2 and 0.70, respectively. Considering the consistency distribution, the consistency threshold was set at 0.80. Table 6 presents the results of the configurations for the absence of radical innovation. The overall consistency was 0.864 and the overall coverage was 0.366. The three configurations share the same core conditions of R&D intensity and explorative learning. The absence of R&D intensity, explorative learning, and exploitative learning appeared in all the solutions; therefore, we performed a supplementary analysis on the necessity of the three conditions for the absence of radical innovation. The results indicated that none of the three conditions alone were a necessary condition for the outcome. The configurations for the absence of SMEs’ radical innovation revealed that whether the external environment is turbulent or not, R&D intensity and explorative learning are important sources of new knowledge to be applied to generate radical innovation. Without high levels of R&D intensity and explorative learning, it is almost impossible to achieve radical innovation (Hill and Rothaermel, 2003).

**TABLE 6:** Configurations for the absence of radical innovation.

Configuration	Solution		
	1a	1b	1c
*Environmental turbulence*			
Technological turbulence	⨂		⨂
Market turbulence		•	•
*Absorptive capacity*			
R&D intensity	⊗	⊗	⊗
Exploratory learning	⊗	⊗	⊗
Transformative learning	⨂	⨂	
Exploitative learning	⨂	⨂	⨂
Consistency	0.837	0.904	0.921
Raw coverage	0.277	0.185	0.185
Unique coverage	0.136	0.044	0.044
Overall solution consistency	0.864		
Overall solution coverage	0.366		

*The black circles* (🌑) *represents the presence of a condition, the crossed-out circle* (⊗) *means the absence of a condition, and the blank space indicates a “don’t care” situation. Large and small circles represent core conditions and peripheral conditions, respectively.*

## Discussion

### Theoretical Contributions

This study has examined the relationship between environmental turbulence, absorptive capacity, and SMEs’ radical innovation, detected several configurations for SMEs to achieve high radical innovation, and made some theoretical contributions as followed. First, we contribute to the research literature on SMEs and radical innovation. We examined how SMEs achieve radical innovation from a configurational perspective and identified several equally effective pathways. In comparison with traditional variance-based methods, such as multiple regression and structural equation modeling, which emphasize the “net effect” between variables, fsQCA focuses on the complex causal relationships through configurational comparative analysis (Ragin and Fiss, 2008). Based on the analysis on real cases rather than the hypothetical-average case, this method can help advance our understanding of complex business phenomena (Douglas et al., 2020). By indicating and comparing the pathways to the presence and absence of high radical innovation, we verify some prior findings on the relationship between environmental turbulence, absorptive capacity, and SMEs’ radical innovation (e.g., Hill and Rothaermel, 2003; Jansen et al., 2006). Besides, although explorative learning is more valued in turbulent environments (Teece, 2007), our results indicate that SMEs should give priority to R&D, transformative learning, and exploitative learning, thus providing some useful insights for SMEs into how to flexibly allocate their resources and capabilities to generate high radical innovation.

Second, we resolve some previously conflicting findings by revealing multiple pathways to high radical innovation. Among these pathways, the underlying conditions can be present, absent, or indifferent, showing that there exist alternative explanations for SMEs to achieve radical innovation. This causal asymmetry of fsQCA is particularly useful for understanding the heterogeneity of business entities and their different ways of surviving and achieving success in turbulent business environments. Previous research has adopted different perspectives and approaches to hypothesize and test the relationship between environmental turbulence, absorptive capacity, and SMEs’ radical innovation and has reported contradictory findings (e.g., Prajogo and McDermott, 2014; Forés and Camisón, 2016; Bodlaj and Cater, 2019). However, while the net effect detected by symmetric methods might be that the independent and dependent variables are positively related, the relationship may be negative or statistically non-significant for a minority of cases within the sample (Douglas et al., 2020). Instead of neglecting the data relationship for these minority groups, identifying the causal asymmetry and investigating these differences will help us resolve those contradictory findings and promote our understanding of the complex causal relationship between environmental turbulence, absorptive capacity, and SMEs’ radical innovation in a more holistic way.

Third, this study also contributes to dynamic capabilities theory and the absorptive capacity literature. As mentioned earlier, few research studies examine the complementary effects of the two dimensions of absorptive capacity (see Carlo et al., 2012 for an exception). We theorized SMEs’ R&D intensity, explorative, transformative, and exploitative learning processes using one model and explored their interdependence with environmental factors when influencing the generation of radical innovation. The results show that R&D intensity can substitute for explorative and transformative learning processes in a relatively stable environment, which is in accordance with previous research on knowledge and radical innovation (Zhou and Li, 2012). Moreover, to address the dynamic environment, firms must balance explorative and exploitative learning (Jansen et al., 2006). The results also indicate the important role of transformative learning in the process of radical innovation. However, this learning process has not received sufficient research attention so far (Marsh and Stock, 2006). Therefore, through a deep look into the interaction between technological and market turbulence, R&D intensity, and the three learning processes, this study extends the research literature on absorptive capacity and dynamic capabilities and provides new insights for future research.

### Practical Implications

This study provides several practical implications for SMEs. First, every firm should develop its own core competence. While some SMEs gain a foothold in the market through imitative innovation, this is not a long-term option. SMEs can only fully take advantage of explorative learning in a stable business environment where trends can easily be recognized. To compete and succeed in today’s dynamic environment, SMEs should invest more resources into their R&D and the establishment and maintenance of their own knowledge base. Second, explorative or exploitative learning can both lead to high radical innovation. This depends on the SMEs’ careful evaluations and choices because explorative learning is neither easy nor costless. When the external environment changes rapidly, especially when technological and market turbulence is at high levels, the potentially valuable external knowledge becomes difficult to recognize and assimilate. Therefore, SMEs should balance exploration and exploitation based on their internal knowledge base and external environmental circumstances. Finally, the transformative learning process should not be ignored because turning nascent technology into marketable products or services is an extensive process. Thus, firms should focus on maintaining their knowledge base over time so that related knowledge can be reactivated and applied when needed.

### Limitations and Future Research

Some limitations of this study should be addressed in future research. First, we used the snowball sampling method, which may limit the generalizability of the findings. However, as indicated by Fiss (2011), the validity of the solutions provided by fsQCA is not threatened by sample representativeness because the results of the truth table analysis are not sensitive to outliers (Pappas and Woodside, 2021). Hence, the findings of this study are relatively robust. However, future studies should use a random sample. Second, the data were collected using self-reported questionnaires and calibrated using the data distribution percentiles. Future research should use more objective data and calibration thresholds. Third, because of the difficulty of data collection, we did not compare the differences between the configurations for large companies’ radical innovation vs. SMEs’ radical innovation. It would be interesting for future studies to examine how large businesses and SMEs achieve the same outcomes through different pathways.

## Conclusion

Drawing on the dynamic capabilities theory, this study has conducted a fuzzy-set qualitative comparative analysis of the relationship between environmental turbulence, absorptive capacity, and SMEs’ radical innovation. The results indicated that the identified causal conditions interacted in a complex way and that different combinations of these conditions can equivalently lead to high radical innovation. Thus, SMEs could achieve radical innovation through flexibly allocating their limited resources to R&D intensity and the three learning processes based on their environmental circumstances. SMEs should prioritize investment in R&D and transformative learning to store technological and market knowledge so that they can later respond quickly to changes. SMEs should also decide whether to explore or exploit depending on the environmental conditions.

## Data Availability Statement

The raw data supporting the conclusions of this article will be made available by the authors, without undue reservation.

## Ethics Statement

The studies involving human participants were reviewed and approved by the Hubei University. The patients/participants provided their written informed consent to participate in this study. Written informed consent was obtained from the individual(s) for the publication of any potentially identifiable images or data included in this article.

## Author Contributions

All authors listed have made a substantial, direct, and intellectual contribution to the work, and approved it for publication.

## Conflict of Interest

The authors declare that the research was conducted in the absence of any commercial or financial relationships that could be construed as a potential conflict of interest.

## Publisher’s Note

All claims expressed in this article are solely those of the authors and do not necessarily represent those of their affiliated organizations, or those of the publisher, the editors and the reviewers. Any product that may be evaluated in this article, or claim that may be made by its manufacturer, is not guaranteed or endorsed by the publisher.

## Appendix

### Items for Causal and Outcome Conditions

a) **Items for radical innovation**
1. We develop products or services that offer greater advantages to customers than any other products or services currently available.
2. We develop products or services that better meet the needs of customers than any other product or service currently available.
3. We develop products or services that require customers to substantially alter their behavior.
4. We introduce new products/services to an existing market.
5. We introduce new products/services to a new market.
6. We develop new product/services that require significantly new technology or ideas that did not exist in the market before.
7. We create new major product/service programs leading to expansion of current markets.
8. We develop innovations that make our prevailing product/service lines obsolete.
9. We introduce new or significantly improved processes for producing or supplying products (goods or delivering services) which are new to our industry.
b) **Items for technological turbulence**
1. The technology in our industry is changing rapidly.
2. Technological changes provide big opportunities in our industry.
3. A large number of new product ideas have been made possible through technological breakthroughs in our industry.
c) **Items for market turbulence**
1. In our kind of business, customers’ product preferences change quite a bit over time.
2. Our customers tend to look for new product all the time.
3. New customers tend to have product-related needs that are different from those of our existing customers.
d) **Items for explorative learning process**
1. We frequently scan the environment for new technologies.
2. We thoroughly observe technological trends.
3. We observe in detail external sources of new technologies.
4. We periodically organize special meetings with external partners to acquire new technologies.
5. Employees regularly approach external institutions to acquire technological knowledge.
6. We often transfer technological knowledge to our firm in response to technology acquisition opportunities.
e) **Items for transformative learning process**
1. We thoroughly maintain relevant knowledge over time.
2. Employees store technological knowledge for future reference.
3. We communicate relevant knowledge across the units of our firm.
4. When recognizing a business opportunity, we can quickly rely on our existing technological knowledge.
5. We quickly analyze and interpret changing market demands for our technologies.
6. New opportunities to serve our customers with existing technologies are quickly understood.
f) **Items for exploitative learning process**
1. We are proficient in transforming technological knowledge into new products.
2. We regularly match new technologies with ideas for new products.
3. We quickly recognize the usefulness of new technological knowledge for existing knowledge.
4. We regularly apply technologies in new products.
5. We constantly consider how to better exploit technologies.
6. It is well known who can best exploit new technologies inside our firm.
